# Mechanisms of Magnesium Transport Through Cell Membranes: The Primacy of the Free Divalent Cation and Implications for Magnesium Supplementation

**DOI:** 10.3390/nu18213651

**Published:** 2026-11-03

**Authors:** Jürgen Vormann, Klaus Kisters, Oliver Micke, Martin Kolisek

**Affiliations:** 1 IPEV Institute for Prevention and Nutrition, 85737 Ismaning, Germany; 2 Operasan Dialysis Center, 44623 Herne, Germany; klauskisters61@gmail.com; 3 Department of Radiotherapy and Radiation Oncology, Franziskus Hospital Bielefeld, 33615 Bielefeld, Germany; strahlenklinik@web.de; 4 Biomedical Centre Martin, Jessenius Faculty of Medicine in Martin, Comenius University in Bratislava, 03601 Martin, Slovakia; martin.kolisek@uniba.sk

**Keywords:** magnesium transport, TRPM6, TRPM7, SLC41A1, CNNM, Mg^2+^ ion, intestinal absorption, bioavailability, magnesium supplementation, organ distribution

## Abstract

Magnesium (Mg^2+^) is the second most abundant intracellular cation and an essential cofactor for more than 600 enzymatic reactions. Commercial claims that particular magnesium salts preferentially deliver magnesium to the brain, heart or muscle raise a mechanistic question: do established transport pathways support selective delivery of an intact magnesium complex? This narrative review examines the dissolution of magnesium salts in the gastrointestinal tract, paracellular and transcellular intestinal absorption, plasma magnesium speciation, the transport and regulatory proteins involved in cellular magnesium handling, and selected formulation-comparison studies. Magnesium is absorbed as the free divalent cation Mg^2+^, and the anion of an ingested salt is handled independently. In plasma, approximately 55% of magnesium is ionised, 30% is bound to albumin and 15% is complexed with small anions. All characterised magnesium transport proteins, including TRPM6, TRPM7, SLC41A1, CNNM proteins, MagT1 and MRS2, handle the free Mg^2+^ ion. The literature reviewed does not establish a pathway that recognises an ingested magnesium–ligand complex and delivers it selectively to a particular organ. Supplements may nevertheless differ in dissolution, gastrointestinal tolerability, systemic exposure and independent effects of the anion. Claims of organ-specific magnesium delivery based on salt form therefore lack a demonstrated mechanistic basis.

## 1. Introduction

Magnesium is the fourth most abundant mineral in the human body and the second most prevalent intracellular cation after potassium. With a total body content of approximately 25 g, it is distributed predominantly in bone (~60%), muscle (~25%), and other soft tissues (~14%), with less than 1% residing in the extracellular fluid and about 0.3% in serum [1,2]. As an obligate cofactor for adenosine triphosphate (ATP), Mg^2+^ participates in virtually all phosphorylation reactions, nucleic acid synthesis, and protein biosynthesis. Over 600 enzymes require Mg^2+^ for catalytic activity [3,4]. Examples include kinases of glycolysis and energy metabolism, such as hexokinase, phosphofructokinase, pyruvate kinase and creatine kinase; ATPases such as the Na^+^/K^+^-ATPase and Ca^2+^-ATPases; and DNA and RNA polymerases [3,5,6].

Because of these fundamental roles, an inadequate magnesium supply has wide-ranging consequences. Overt hypomagnesaemia (serum magnesium below approximately 0.7 mmol/L) manifests as neuromuscular hyperexcitability (tremor, muscle cramps, tetany, seizures), cardiac arrhythmias including torsades de pointes, and secondary hypocalcaemia and hypokalaemia resulting from impaired parathyroid hormone secretion and action and from renal potassium wasting [1,5]. Chronic latent magnesium deficiency, for which a serum magnesium threshold of approximately 0.85 mmol/L has been proposed, is far more common and frequently goes undetected because serum magnesium is maintained for long periods at the expense of intracellular and bone stores. In epidemiological and clinical studies, it has been associated with insulin resistance and type 2 diabetes, hypertension, cardiovascular disease, osteoporosis, migraine, and depression [4,6,7,8]. Iatrogenic causes, including long-term use of proton-pump inhibitors, diuretics, calcineurin inhibitors, and antibodies targeting the epidermal growth factor receptor (EGFR), add further to the burden of deficiency [5,9,10]. Correcting magnesium deficiency efficiently is therefore of considerable clinical and public-health relevance.

Despite its biological importance, insufficient magnesium intake is common. In Western populations, dietary intake frequently falls below the Recommended Dietary Allowance (RDA) of 310–420 mg/day for adults, prompting widespread use of oral supplementation [6,11]. In the United States, nearly half of the population is estimated to consume less than the Estimated Average Requirement [8], and in Spain, about 75% of the population has intakes below 80% of national and European recommendations [6]. Supplementation at usual doses is considered safe. The tolerable upper intake level (UL) refers only to magnesium from supplements and pharmaceutical preparations. It is set at 250 mg/day by the Scientific Committee on Food of the European Commission and at 350 mg/day by the US Institute of Medicine [6,12,13]. In both cases, it is derived from data on mild and transient osmotic diarrhoea rather than systemic toxicity, and it does not apply to magnesium given for medical treatment under medical supervision [13]. The UL is a scientific reference value. Legal maximum amounts for food supplements have not yet been harmonised in the European Union [14], and products with higher doses are frequently authorised as medicinal products. In a recent analysis of randomised trials and meta-analyses, seven of ten studies with supplemental doses of 128 to 1200 mg/day found no significant difference in diarrhoea between magnesium and control groups, and the authors called for a re-evaluation of the UL [15]. The market offers a wide array of formulations, including oxide, citrate, chloride, lactate, glycinate, taurate, malate, orotate and threonate, alone or in combination. Many are marketed with claims of superior bioavailability or, more specifically, preferential delivery of magnesium to particular organs such as the brain, heart or muscle.

The central question of this review is whether the established mechanisms of magnesium transport support such organ-specific delivery. We examine the transport-based rationale and the limits of the evidence. An exhaustive assessment of commercial products or clinical efficacy is beyond the scope of this review.

To address this question, this narrative review examines: (1) magnesium salt dissolution and luminal speciation; (2) paracellular and transcellular intestinal absorption; (3) plasma magnesium fractions and their equilibration; (4) functional and structural evidence for selected mammalian magnesium transport and regulatory proteins; (5) tissue-specific regulation of magnesium homeostasis; and (6) studies reporting formulation-dependent tissue or functional outcomes.

### Literature Identification and Scope

This is a focused narrative review, not a systematic review. Targeted searches of PubMed, PubMed Central full texts and publisher platforms were performed, most recently in October 2026. Terms combined magnesium absorption, bioavailability, speciation, salt or formulation with TRPM6, TRPM7, SLC41, CNNM, MAGT1, TUSC3, MRS2, claudin, tissue distribution or clinical trial; author/title searches were used to check the cited studies. Reference lists of relevant reviews and primary studies were followed to foundational work, without a lower publication-date limit. Selection prioritised English-language primary transport, structural, genetic and absorption studies, authoritative physiological reviews, and selected animal and human formulation comparisons relevant to tissue exposure or functional outcomes. Commercial claims were not treated as efficacy evidence. Study model, measured magnesium endpoint and direct versus inferred mechanism were considered during interpretation. The clinical examples are illustrative rather than an exhaustive comparative efficacy assessment; no protocol, duplicate screening or formal risk-of-bias assessment was used. Negative mechanistic statements therefore refer to the literature considered, not proof that every possible pathway has been excluded.

## 2. Magnesium in the Gastrointestinal Tract: Dissociation and Luminal Chemistry

### 2.1. Dissociation of Magnesium Salts

Dissolution of inorganic salts and dissociation of organic magnesium complexes make Mg^2+^ available to ion-conducting pathways in the intestinal epithelium. Luminal magnesium speciation depends on pH, solubility, ligand concentrations and the food matrix [9,16]. Schuchardt and Hahn state that magnesium is generally absorbed as the ion, but also that it is not known whether magnesium may be absorbed together with other nutrients or in the form of complexes [16]. The present review examines this open question from the perspective of the transport proteins involved. The degree and rate of dissociation depend primarily on the physicochemical properties of the salt, principally its solubility in the acidic gastric environment (pH 1.5–3.5) and the more neutral intestinal milieu (pH 6–7.5). Organic magnesium salts, which are generally more water-soluble than poorly soluble inorganic forms such as magnesium oxide (solubility ~0.009 g/100 mL) or carbonate, dissociate more readily and completely, yielding higher concentrations of free Mg^2+^ ions in the intestinal lumen [16,17]. Magnesium in mineral water is already dissolved, and its absorption is further enhanced when the water is consumed with a meal or in several servings over the day [16].

### 2.2. Gastric Acid and the Role of Luminal pH

Gastric acid plays a crucial role in liberating Mg^2+^ from dietary and supplemental sources. The acidic gastric environment protonates the anion ligands of organic salts and promotes dissolution of inorganic salts, including the otherwise poorly soluble magnesium oxide. Gastric acid suppression, for example, by proton-pump inhibitors (PPIs), may impair this dissolution step. PPIs are also thought to reduce intestinal magnesium transport itself, and both effects may contribute to clinically relevant hypomagnesaemia [9,18].

Luminal bicarbonate secretion, conversely, can precipitate Mg^2+^ as insoluble MgCO_3_, thereby reducing the available free ion pool. This is consistent with the view that magnesium must be in solution as the free ion to be absorbed [9,19]. Further dietary factors modify luminal availability. Phytate lowers magnesium absorption in a dose-dependent manner, and absorption from oxalate-rich foods such as spinach is lower than from low-oxalate foods. A higher protein intake increased magnesium absorption in human studies, and low- or indigestible carbohydrates such as resistant starch, oligosaccharides and inulin enhanced it, predominantly in animal studies [16].

### 2.3. Salt Form, Solubility, and Fractional Absorption

Studies comparing magnesium bioavailability across salt forms in vitro and in humans show that differences, where they exist, are modest and can largely be explained by differences in solubility [16,20]. These studies use different models that are not interchangeable. In vitro dissolution and simulated digestion assess bioaccessibility, Caco-2 monolayers assess cellular transport, and animal and human studies measure serum, urinary or tissue magnesium after a defined dose. Fractional absorption in humans also declines with increasing dose, which complicates comparisons between products [16,21]. In a Caco-2 monolayer model, Kyselovič et al. tested oxide, sulphate, chloride, carbonate, lactate, citrate and pidolate. At the tested equimolar magnesium concentrations, the authors reported significantly lower absorption for citrate, chloride and sulphate than for carbonate, pidolate and oxide after 15 min and 2 h [22]. Culture-medium conditions, limited physiological realism and the unexpectedly favourable oxide result restrict extrapolation to human absorption; the authors themselves call for in vivo validation [22].

Blancquaert et al. found substantial differences in dissolution and simulated gastrointestinal availability among 15 commercial formulations. Subsequent testing in 30 healthy adults showed greater acute serum magnesium exposure with a magnesium oxide/glycerophosphate formulation than with an oxide formulation, despite the latter providing more than twice as much elemental magnesium (450 versus 196 mg) [20]. This supports a contribution of formulation to systemic availability. The human study measured serum and urinary magnesium over a short interval and did not measure organ distribution. Fine et al. found that fractional absorption declined with increasing intake and that magnesium from almonds was absorbed similarly to soluble magnesium acetate under their test conditions [21]. Neither study identified the molecular species traversing the epithelium.

Magnesium lactate, which is widely used in medicinal products and supplements, is readily soluble. In healthy volunteers, its bioavailability, measured as the increase in urinary magnesium excretion, was significantly higher than that of magnesium oxide and equivalent to that of magnesium chloride and magnesium aspartate [23]. As lactate is an endogenous metabolite, these findings reflect differences in luminal availability rather than a specific action of the anion.

## 3. Intestinal Magnesium Absorption: Paracellular and Transcellular Pathways

Intestinal magnesium absorption includes a concentration-dependent passive paracellular route and a saturable transcellular route involving TRPM6/TRPM7-containing channels [9,16]. The channels conduct Mg^2+^, and ionic flux is the established model for these pathways. Their relative contributions vary with intake, intestinal segment and experimental system [2,16].

### 3.1. Paracellular Transport: Claudin-Mediated Passive Diffusion

Paracellular transport is an important route of intestinal magnesium absorption, particularly at higher luminal magnesium concentrations. Estimates of approximately 80–90% are often cited, but they should not be treated as a fixed proportion across intakes and intestinal segments [9,16]. Flux depends on the electrochemical gradient and epithelial permeability; the balance with saturable transcellular transport changes as intake changes [2,21].

The molecular conduits for paracellular Mg^2+^ flux are members of the claudin family of tight junction proteins. Claudin-7 (CLDN7) and claudin-12 (CLDN12) have been implicated in paracellular Mg^2+^ permeability of the small intestine [9,18]. The selectivity of claudin pores is based on ionic charge and hydrated radius, and no recognition site for an organic ligand has been described [24].

Mechanistically, claudins are tetraspan membrane proteins whose two extracellular loops interact with claudins of the adjacent cell to form the tight-junction strands. The first extracellular loop (ECL1) lines a narrow, water-filled paracellular pore, and the number and position of charged amino acid residues within ECL1 determine its charge selectivity: negatively charged residues create an electrostatic environment that favours the passage of cations, whereas positively charged residues favour anions [24]. Cations move through these pores passively, down the electrochemical gradient generated by the lumen-positive transepithelial voltage and the luminal-to-interstitial concentration difference, and partly by solvent drag with absorbed water [9,24]. Unlike transcellular channels, paracellular claudin pores do not require complete dehydration of the permeating ion; Mg^2+^ is thought to traverse them in a hydrated or partially hydrated state so that selectivity is determined by charge and hydrated size rather than by specific coordination chemistry [24].

Claudin-2 and claudin-12 form such cation-selective pores in the intestine and are required for vitamin D-dependent paracellular Ca^2+^ absorption [25]; by analogy, and supported by functional data, they are considered to contribute to paracellular Mg^2+^ flux [9]. The evidence for claudin-7 and claudin-12 specifically derives largely from Caco-2 monolayers. In this model, omeprazole suppressed passive Mg^2+^ transport, lowered the apparent Mg^2+^ affinity of the paracellular pathway, and decreased claudin-7 and claudin-12 (but not claudin-2) expression in a concentration-dependent manner, whereas apical acidification increased claudin-7 and claudin-12 expression and abolished the inhibitory effect of omeprazole on Mg^2+^ transport [18]. These data establish a close association between claudin-7/-12 abundance and paracellular Mg^2+^ permeability. Direct proof that these proteins constitute the Mg^2+^-permeable pore, for example, from intestine-specific knockout models or reconstitution studies, is still lacking, and claudin-7 has been described as a barrier-forming rather than a pore-forming claudin in some other epithelia [24]. Irrespective of the precise molecular composition of the pore, neither the electrostatic selectivity filter formed by ECL1 nor the dimensions of the paracellular pathway provide a recognition site for an organic magnesium–ligand complex.

Notably, the relative expression of ‘tightening’ claudins 1, 3, 4, 5 and 8, which reduce paracellular permeability, is lower in the small intestine than in other epithelia, thereby permitting the passive diffusion of divalent cations including Mg^2+^ [9,16]. Renal claudin-16 (paracellin-1) and claudin-19 perform an analogous role in the thick ascending limb of the loop of Henle, where mutations cause familial hypomagnesaemia with hypercalciuria and nephrocalcinosis [19,26].

### 3.2. Transcellular Transport: TRPM6 and TRPM7

Transcellular absorption occurs mainly in the distal small intestine and colon, where Mg^2+^ enters the enterocyte through apical channels formed by TRPM6 and TRPM7 [16,27]. Channel activity is tightly regulated by intracellular free Mg^2+^ and by MgATP. When intracellular Mg^2+^ rises, TRPM7 is inhibited, which limits further entry. Whether TRPM6 forms functional homomeric channels at the plasma membrane is debated [27,28]. Functional epithelial transport appears to rely largely on heteromeric TRPM6/TRPM7 channels, which are less sensitive to inhibition by intracellular Mg^2+^ and MgATP than TRPM7 homomers [28,29]. This allows continued epithelial Mg^2+^ uptake across the usual range of dietary intake, while feedback inhibition still protects the cell against intracellular overload [28,29].

### 3.3. Basolateral Extrusion and Charge Balance

Following apical entry via TRPM6/7, Mg^2+^ must be extruded across the basolateral membrane to enter the interstitial fluid and portal circulation. Basolateral extrusion is thought to be mediated mainly by CNNM4, with a possible contribution from SLC41A1 [30,31].

**Figure 1:**
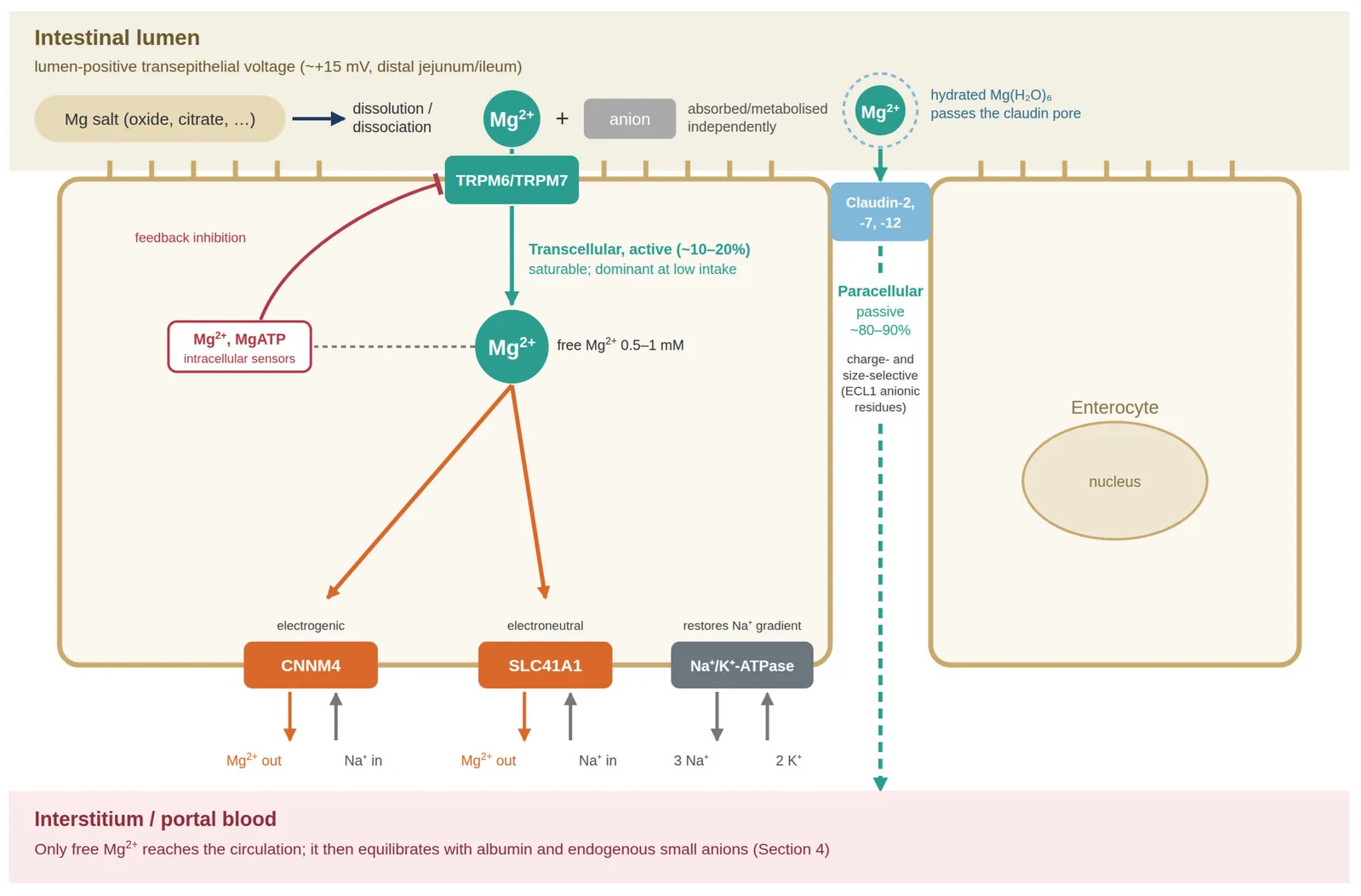
Intestinal absorption of magnesium. Magnesium salts dissolve and dissociate in the intestinal lumen, releasing free Mg^2+^ and the respective anion, which is handled independently. Free Mg^2+^ is absorbed either passively through cation-selective claudin pores of the tight junction (paracellular pathway, quantitatively predominant at higher luminal concentrations) or actively via apical TRPM6/TRPM7 channels (transcellular pathway), whose activity is limited by intracellular free Mg^2+^ and MgATP. Basolateral extrusion is attributed to CNNM4 and SLC41A1, probably coupled to Na^+^ entry. The Na^+^ gradient is maintained by the Na^+^/K^+^-ATPase. Only free Mg^2+^ reaches the portal circulation through these pathways. Based on references [9,16,18,24,25,28,30,31].

Both proteins have been proposed to couple Mg^2+^ extrusion to Na^+^ entry. Na^+^-dependent Mg^2+^ extrusion has been reported for CNNM4 and SLC41A1 in cell-expression studies [30,31]. However, Arjona et al. observed SLC41A1-mediated ^25^Mg extrusion in HEK293 cells that persisted after replacement of extracellular Na^+^, and they did not identify the counter-ion or a complete transport mechanism [32]. Strict Na^+^ dependence, electroneutrality and a fixed Na^+^:Mg^2+^ stoichiometry should therefore not be regarded as established for SLC41A1. Whether CNNM proteins mediate Mg^2+^ efflux themselves or act mainly as regulators also remains debated, and the available evidence does not establish a fixed Na^+^:Mg^2+^ exchange ratio for CNNM4 [33,34]. Where extrusion is Na^+^-coupled, the inward Na^+^ gradient is maintained by the basolateral Na^+^/K^+^-ATPase [5,35]. The complete intestinal absorption pathway is summarised in Figure 1.

## 4. Magnesium in the Bloodstream: Speciation Independent of the Original Salt Form

After absorption, circulating magnesium partitions among ionised, protein-bound and small-anion-complexed fractions (Table 1).

In plasma, total magnesium exists in three distinct fractions [36,37]. The free ionised Mg^2+^ (iMg^2+^) fraction, approximately 55% of total plasma magnesium, constitutes the biologically active pool and the substrate for cellular uptake by all known magnesium transport proteins. Scarpati et al. [36] emphasize that iMg^2+^ is the only fraction that specifically identifies true dysmagnesemia and directly reflects cellular availability. Total serum magnesium, which includes protein-bound and complexed fractions, does not reliably reflect intracellular concentrations or tissue availability [36,38].

The protein-bound fraction (~30%) is principally bound to albumin through non-covalent interactions; this fraction is in dynamic equilibrium with the free pool but is not directly transported into cells. The anion-complexed fraction (~15%), formed with endogenous plasma anions such as citrate, phosphate, bicarbonate and hydroxide, is also in equilibrium with free iMg^2+^. For example, absorbed citrate participates in the same chemistry as endogenous citrate, but circulating concentrations and biological effects may still depend on intake and metabolism [16,36].

This speciation profile is maintained by concentration-dependent equilibria and binding affinities. In most organs, including the heart, liver, kidney and skeletal muscle, the iMg^2+^ concentration of the interstitial fluid follows plasma iMg^2+^. The central nervous system is an exception because the blood–brain and blood–CSF barriers regulate magnesium entry (Section 7.1). No mechanism is known by which the original supplemental salt form could alter this distribution once magnesium has been absorbed [3,38].

**Functional significance of the individual magnesium fractions.** The three plasma fractions differ not only in abundance but also in function. (i) Free ionised Mg^2+^ is the only fraction that interacts directly with ion channels, receptors, and transporters. It is the species that antagonises Ca^2+^ entry through voltage-gated Ca^2+^ channels, stabilises excitable membranes by screening negative surface charges, is sensed by the Ca^2+^/Mg^2+^-sensing receptor in the parathyroid gland and kidney, and serves as the substrate for all cellular uptake pathways [3,5,36]. The physiological consequences of hypo- and hypermagnesaemia are therefore mediated through the free fraction. (ii) The protein-bound fraction acts as a circulating buffer and reservoir. Binding to albumin is pH-dependent: as pH rises, ionised Mg^2+^ falls because protons compete with divalent cations for binding sites on albumin [39]. In hypoalbuminaemia, total serum magnesium may be low while iMg^2+^ remains normal, which is one reason why total magnesium can misclassify magnesium status [36,38]. Protein-bound magnesium is not filtered by the glomerulus (Section 6) and is not transported into cells. (iii) The complexed fraction, formed with citrate, phosphate, bicarbonate, sulphate, and lactate, is ultrafilterable and contributes to the filtered renal load. Its size varies with the plasma concentrations of these endogenous anions; it has no known signalling function and dissociates rapidly to replenish the free pool [36,37].

Inside cells, magnesium likewise exists in different forms with distinct functions. The majority of intracellular magnesium is bound to ATP and other nucleotides, and MgATP, rather than free ATP, is the true substrate of virtually all kinases and ATPases, including the Na^+^/K^+^-ATPase [3,5]. Further large fractions are bound to ribosomes and nucleic acids, where Mg^2+^ stabilises RNA and DNA structure and is required for ribosome assembly and translation, and to membrane phospholipids. Only the small free fraction (0.5–1.0 mM) acts as a regulatory signal, for example, by modulating the gating of TRPM7, TRPM6, and MRS2 and by allosterically regulating enzymes [3,28,40].

With respect to supplements, the commercial ‘forms’ of magnesium (oxide, citrate, glycinate, taurate, threonate, and others) differ in solubility, elemental magnesium content, and gastrointestinal tolerability, and some of their anions have independent pharmacological actions (Section 7.6 and Section 10). They do not, however, give rise to different forms of magnesium within the body.

## 5. Molecular Mechanisms of Cellular Magnesium Transport

Mammalian cells maintain intracellular free Mg^2+^ concentrations in the range of 0.5–1.0 mM in the presence of a total cellular magnesium content of 15–20 mM (the majority of which is complexed with ATP, nucleic acids, and phospholipids) [3,40]. This regulated equilibrium is achieved through the coordinated activity of several distinct membrane transport proteins, all of which transport only the free Mg^2+^ ion.

### 5.1. TRPM7: The Ubiquitous Cellular Magnesium Gateway

TRPM7 is expressed in virtually every mammalian cell type and is considered the primary pathway for Mg^2+^ entry across the plasma membrane of non-epithelial cells. Zou et al. [41] review TRPM7 function in detail. The channel conducts Mg^2+^, Ca^2+^ and Zn^2+^, and permeation of magnesium–amino acid or magnesium–organic acid complexes has not been reported.

TRPM7 forms functional homotetramers. Its activity is inhibited by intracellular free Mg^2+^ and MgATP and increases when intracellular Mg^2+^ falls. This homeostatic feedback limits the accumulation of excess magnesium. Growth factors, including EGF and VEGF, regulate TRPM7 activity via receptor tyrosine kinase signaling pathways [41]. None of these regulatory inputs is known to depend on the chemical form of ingested magnesium. At the extracellular face of the membrane, the channel encounters free iMg^2+^.

**Figure 2:**
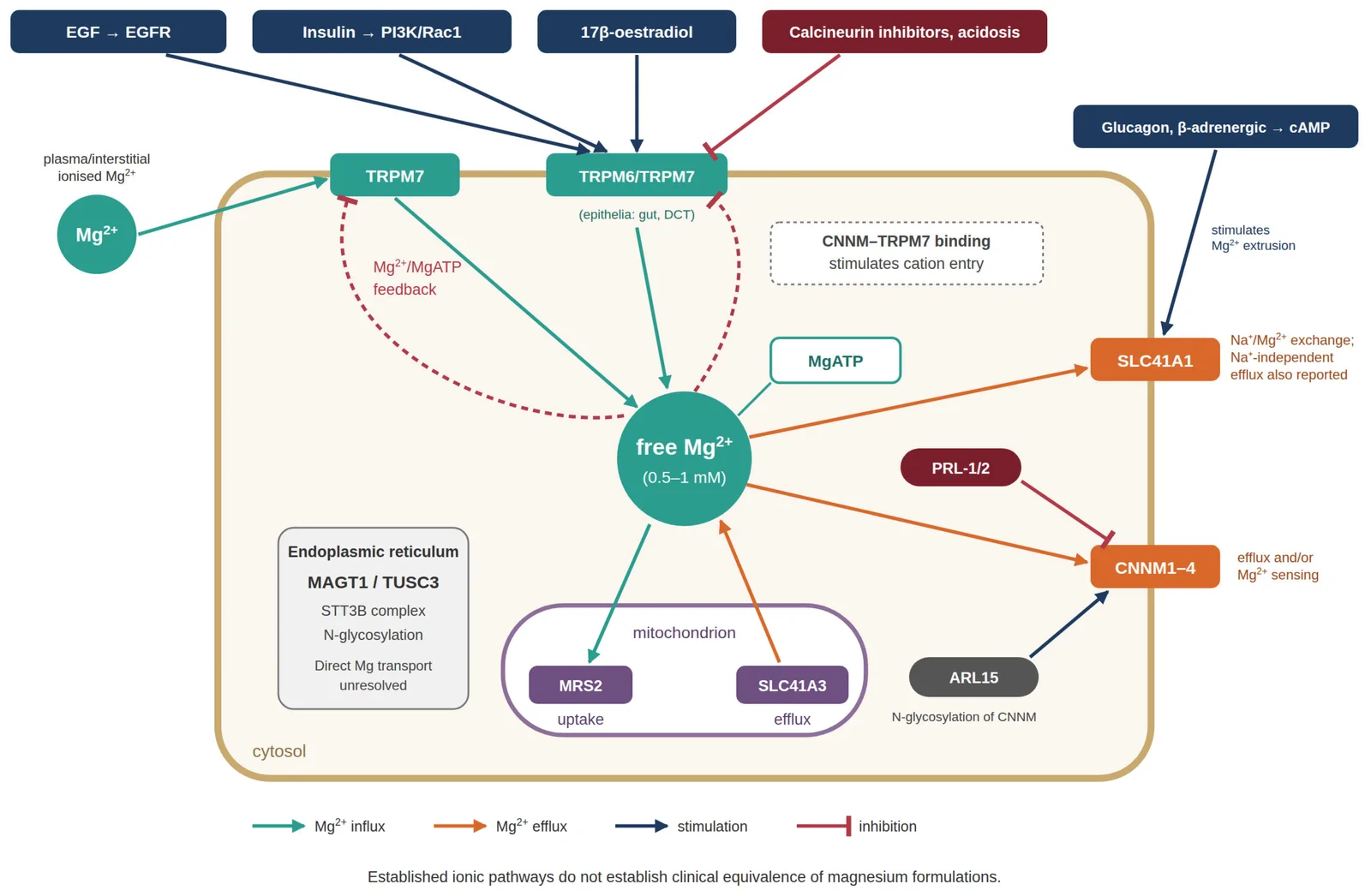
Network of proteins involved in cellular Mg^2+^ homeostasis. Free extracellular Mg^2+^ enters cells primarily via TRPM7 (ubiquitous), TRPM6/TRPM7 heteromers (epithelia), and MagT1. Intracellular free Mg^2+^ (0.5–1 mM) is in equilibrium with MgATP and other bound forms; it is taken up into mitochondria by MRS2 and released from mitochondria by SLC41A3. Extrusion across the plasma membrane is mediated by SLC41A1 (Na^+^/Mg^2+^ exchange) and CNNM proteins. Intracellular Mg^2+^ and MgATP inhibit TRPM6/7 and MRS2 (feedback). PRL-1/2 binds CNNM proteins and inhibits Mg^2+^ efflux; ARL15 modulates CNNM function via *N*-glycosylation; CNNM proteins also bind TRPM7. Extracellular regulators include EGF, insulin, and 17β-oestradiol (stimulating TRPM6); calcineurin inhibitors and acidosis (inhibiting TRPM6); and glucagon/β-adrenergic agonists acting via cAMP to stimulate Mg^2+^ extrusion. Based on references [4,10,28,29,31,32,41,44,66,67,68,69,70].

It should be emphasised that TRPM7 does not itself remove excess Mg^2+^ from cells or from the body. Although TRPM7 currents show pronounced outward rectification, the outward current at positive membrane potentials is carried mainly by monovalent cations. At physiological, inside-negative membrane potentials (approximately −60 to −80 mV), and with extracellular and intracellular free Mg^2+^ concentrations of similar magnitude, the electrochemical gradient for Mg^2+^ is directed into the cell, so TRPM7 can only mediate Mg^2+^ influx. Its contribution to preventing magnesium overload is indirect: as intracellular free Mg^2+^ and MgATP rise, they inhibit the channel and thereby limit further entry [28,29,41]. Active removal of Mg^2+^ from the cytosol against its electrochemical gradient requires energy and is accomplished by secondary active transporters, chiefly SLC41A1 (proposed Na^+^/Mg^2+^ exchange) and the CNNM proteins, which are thought to use the inwardly directed Na^+^ gradient maintained by the Na^+^/K^+^-ATPase (Section 5.2 and Section 5.3) [30,31]. At the whole-body level, excess magnesium is eliminated by the kidney. When plasma Mg^2+^ rises, activation of the Ca^2+^/Mg^2+^-sensing receptor in the thick ascending limb reduces paracellular reabsorption, and TRPM6-mediated reabsorption in the distal convoluted tubule is downregulated so that excess filtered Mg^2+^ is excreted in the urine [2,5,19]. Unabsorbed oral magnesium is excreted in the faeces. The interplay between influx and efflux pathways and their regulators is summarised in Figure 2 and Table 2.

The physiological indispensability of TRPM7 is confirmed by the lethal phenotype of global TRPM7 knockout mice and the essential requirement of intestinal TRPM7 for postnatal mineral homeostasis [42]. The TRPM7 kinase domain, while not required for Mg^2+^ transport per se, modulates channel regulation and intracellular signalling [40,42].

### 5.2. SLC41A1 and SLC41A3: Na^+^/Mg^2+^ Exchangers

The SLC41 family represents the eukaryotic homologues of the prokaryotic MgtE magnesium transporter. Three mammalian members have been characterized: SLC41A1 and SLC41A2 at the plasma membrane and SLC41A3 predominantly in mitochondria [32,33,43].

SLC41A1 is considered primarily a Mg^2+^ efflux transporter. Kolisek et al. [43] showed that heterologous expression of SLC41A1 in Salmonella typhimurium strains deficient in all native Mg^2+^ transporters rescued bacterial growth under Mg^2+^-depleted conditions, demonstrating bona fide Mg^2+^ transport activity. Na^+^-dependent Mg^2+^ extrusion by SLC41A1 was later described in HEK293 cells [31], although Na^+^ dependence has been questioned (see below). SLC41A1 transports Mg^2+^ and, to a lesser extent, other divalent cations such as Zn^2+^, Fe^2+^ and Cu^2+^. Transport of intact organometallic complexes has not been reported [33,43].

In the kidney, SLC41A1 is highly expressed in the distal convoluted tubule and has been proposed to contribute to renal Mg^2+^ reabsorption. Zebrafish knockdown models suggest that SLC41A1 contributes to Mg^2+^ homeostasis in vivo [32]. A more recently characterized DCT-specific isoform of SLC41A3 has been shown to mediate both Mg^2+^ uptake and extrusion independently of Na^+^ and of TRPM7, further expanding the repertoire of cellular Mg^2+^ transport mechanisms [44].

These studies, however, need to be interpreted with caution, and some of their findings are not fully consistent. First, a substantial part of the functional evidence for SLC41A1 was obtained in heterologous systems, namely complementation of Mg^2+^-transport-deficient *Salmonella* strains [43] and overexpression in HEK293 cells [31,32]. In such systems, expression levels, membrane targeting, and the cellular environment differ from native tissues; they can show that a protein is capable of mediating Mg^2+^ flux, but not that it does so in vivo. Second, the ion coupling of SLC41A1 remains controversial. Kolisek et al. characterised SLC41A1 as a Na^+^/Mg^2+^ exchanger [31], whereas Arjona et al. observed Na^+^-independent Mg^2+^ extrusion in HEK293 cells and proposed that SLC41A1 may mediate both uptake and extrusion [32]. Differences in experimental conditions, such as the Na^+^ substitution protocol, Mg^2+^ loading of the cells, and the fluorescent indicators or isotopes used, probably contribute to these discrepancies.

Third, the in vivo relevance of SLC41A1 differs between species. In zebrafish larvae, morpholino knockdown of *Slc41a1* caused renal Mg^2+^ wasting and reduced whole-body magnesium [32], whereas *Slc41a1* knockout mice showed normal serum and urinary Mg^2+^ on both normal and low-magnesium diets, without compensatory changes in renal expression of *Trpm6*, *Cnnm2*, or *Slc41a3* [45]. Possible explanations include species differences in renal magnesium handling, functional redundancy with CNNM proteins, compensation at the protein or post-translational level, and the known limitations of morpholino-based knockdown. Notably, in the same mouse study, *Slc41a3* knockout lowered serum Mg^2+^ by approximately 17%, suggesting that SLC41A3 may be the more relevant family member for systemic homeostasis [45]. Future work should therefore include tissue-specific and inducible knockout models, direct transport measurements with stable isotopes (^25^Mg, ^26^Mg) in native cells, high-resolution structures of SLC41 proteins to resolve stoichiometry and ion coupling, and analysis of human genetic variants. For the question addressed in this review, these open issues do not alter the main conclusion: in every experimental system studied, the transported substrate was the free Mg^2+^ ion.

### 5.3. CNNM Proteins: Mg^2+^ Transporters and Sensors

The CNNM (Cyclin M) family of proteins, CNNM1 to CNNM4, are evolutionarily conserved magnesium transport mediators with structural homology to the prokaryotic CorB/C proteins [33,46]. Their precise molecular function remains subject to debate: some evidence supports a direct Na^+^/Mg^2+^ exchange function, while other studies propose a Mg^2+^ sensor or regulatory role rather than direct transport [33,34].

CNNM2 is highly expressed in the kidney (basolateral membrane of the DCT), and mutations cause severe hypomagnesaemia with seizures and intellectual disability [46]. CNNM4 is expressed in intestinal epithelial cells, and its mutations cause Jalili syndrome [30,34]. In structural terms, the CBS domains of CNNM proteins bind MgATP and free Mg^2+^ ions, inducing conformational changes that regulate the putative transport function [46]. These binding sites accommodate the free Mg^2+^ ion, not an organic magnesium complex.

Regardless of the precise transport versus sensor debate, all functional evidence for CNNM proteins involves the free Mg^2+^ ion as the sole relevant ligand. No study has reported CNNM-mediated transport of any magnesium–organic acid or magnesium–amino acid complex [33,34,46].

### 5.4. MagT1 (MAGT1): Magnesium Transporter 1

MagT1 (encoded by the MAGT1 gene) was characterised as a mammalian plasma membrane protein required for cellular Mg^2+^ uptake [47]. MagT1 is glycosylated and localizes to the plasma membrane. Its transport activity is selective for Mg^2+^ over other divalent cations. More recently, MagT1 and its paralogue TUSC3 have been recognised as subunits of the oligosaccharyltransferase complex. Their roles extend to *N*-glycosylation and immune cell function, and whether MagT1 is itself a direct Mg^2+^ transporter is debated [26].

In cancer biology, TRPM7 overexpression and MagT1 upregulation have been associated with chemoresistance, and altered Mg^2+^ homeostasis mediated by these transporters has been proposed to contribute to drug resistance [48]. MagT1-mediated transport has been demonstrated using ^25^Mg^2+^ isotope tracing and fluorescent Mg^2+^ indicators, consistent with transport of the free ionic form [47].

### 5.5. MRS2: Mitochondrial Magnesium Transport

The mitochondrial RNA splicing protein 2 (MRS2) is the eukaryotic homologue of the prokaryotic CorA magnesium channel family and is the principal transporter responsible for Mg^2+^ uptake into the mitochondrial matrix [26,33]. MRS2 forms pentameric channel complexes in the inner mitochondrial membrane and mediates Mg^2+^ entry driven by the mitochondrial membrane potential.

The structural mechanism of CorA/MRS2-family transporters has been extensively characterized. Guskov et al. [49] solved the crystal structure of CorA, showing that polar residues within the channel coordinate a partially hydrated Mg^2+^ ion during transport. The channel architecture provides a selectivity filter adapted to the hydrated Mg^2+^ ion, which has the smallest ionic radius but the largest hydrated radius of the physiological metal cations [50,51]. A chelated or complexed magnesium species does not fit this coordination geometry.

Intracellular Mg^2+^ sensing by the cytoplasmic *N*-terminal domain of MRS2 regulates channel gating: high cytoplasmic Mg^2+^ stabilizes a closed conformation, while depletion allows channel opening [33]. This autoregulation operates solely on the concentration of free cytoplasmic Mg^2+^.

### 5.6. Biophysical Mechanisms of Ion Selectivity and Dehydration Constraints

Across all mammalian Mg^2+^ transport systems, ion selectivity is dictated by thermodynamic coordination chemistry rather than a simple steric exclusion sieve [50]. The free magnesium ion possesses a very small unhydrated ionic radius (0.72 Å) but carries a rigid, highly stable primary hydration shell of six water molecules, yielding a large hydrated radius of ~4.3 Å [50,51]. The hexahydrated ion (Mg(H_2_O)_6_^2+^) has an effective molecular mass of approximately 132 Da. While an intact magnesium glycinate complex possesses a molecular mass of ∼172 Da, its exclusion from the transporter pore is not driven by raw bulk, but by the high energy barrier of ligand exchange [52,53].

To traverse an ion-selective pore, the Mg^2+^ ion must be at least partially stripped of its hydration shell [54]. Transporter selectivity filters, such as the highly conserved pentameric ring of carboxylate residues in the CorA/MRS2 family or the glutamate motifs in TRPM6/7, are arranged to bind the hydrated ion and to replace its water ligands stepwise as it passes through the pore [54,55].

Magnesium complexes with organic ligands such as glycinate, citrate or threonate have only low to moderate stability constants and dissociate to a large extent at physiological concentrations and pH [16,50]. An intact complex would differ from hydrated Mg^2+^ in size, charge distribution and coordination geometry, and the selectivity filters described above offer no binding mode for it. Translocation of an intact magnesium–ligand chelate through a known mammalian Mg^2+^-selective channel is therefore implausible on both structural and energetic grounds.

The principal mammalian Mg^2+^ transport and regulatory proteins discussed in this section, together with their localisation, transport direction, regulators, and loss-of-function phenotypes, are summarised in Table 2.

## 6. Renal Ultrafiltration Dynamics

Renal magnesium handling is a major determinant of whole-body balance. Approximately 70–80% of circulating magnesium is ultrafilterable: ionised magnesium and small soluble complexes contribute to this pool, whereas albumin-bound magnesium is largely retained [2,26,37]. Most filtered magnesium is reabsorbed along the nephron, and regulated reabsorption permits adaptation of urinary excretion to intake and physiological need [2,19]. Circulating magnesium fractions should therefore be distinguished from the magnesium subsequently transported across renal epithelia.

## 7. Organ-Specific Cellular Magnesium Uptake: Regulation Independent of Salt Form

Commercial claims of preferential magnesium delivery to brain, heart or muscle should be assessed at three distinct levels: selective transport of an intact magnesium complex, differences in systemic magnesium exposure and tissue handling, and clinical outcomes. The first requires a demonstrated molecular delivery mechanism; the other two can vary without intact-complex transport. The tissue summaries below describe physiological regulation, not proof that every formulation has identical effects.

### 7.1. Brain

Magnesium availability in the CNS is constrained by the blood–brain barrier and the blood–CSF barrier at the choroid plexus. Ghabriel and Vink describe evidence for regulated magnesium entry, including transport against a concentration gradient in choroid-plexus preparations, but emphasise that the molecular routes remain incompletely defined. TRPM7 expression in brain endothelial preparations suggests a possible contribution [56]. In 30 patients with acute brain injury, McKee et al. measured baseline total and ionised CSF magnesium of 1.25 ± 0.14 and 0.80 ± 0.10 mmol/L, respectively, compared with serum values of 0.92 ± 0.18 and 0.63 ± 0.07 mmol/L [57]. Intravenous magnesium sulphate produced only modest CSF increases despite marked serum elevation; the study neither compared oral salt formulations nor identified the responsible transporter [57]. Formulation-dependent CNS exposure therefore remains an empirical question distinct from an intact-complex delivery mechanism.

### 7.2. Heart

TRPM7 is considered a major Mg^2+^ entry pathway in cardiomyocytes [41]. Cardiac Mg^2+^ homeostasis is critical for maintaining normal electrophysiology, as Mg^2+^ antagonizes calcium channels and modulates cardiac conduction. Fritzen et al. [7] review the cardiometabolic roles of Mg^2+^, emphasizing that cellular uptake is regulated by transporter expression and intracellular Mg^2+^ sensing.

### 7.3. Skeletal Muscle

Skeletal muscle cells, among the largest intracellular Mg^2+^ reservoirs in the body (~25% of total body Mg^2+^), are thought to rely on TRPM7 for basal Mg^2+^ influx and on SLC41A1 and CNNM proteins for efflux. Muscle Mg^2+^ content is influenced by training status, hormonal milieu (insulin, adrenaline), and overall magnesium intake [3].

### 7.4. Liver

The liver is exposed to portal blood following intestinal absorption and is therefore the first major organ to encounter absorbed magnesium. Hepatocytes regulate intracellular Mg^2+^ through hormone-sensitive transport: glucagon and β-adrenergic stimulation activate Mg^2+^ extrusion from hepatocytes, whereas insulin promotes retention [3]. These hormonal effects are mediated by cAMP-dependent signaling cascades that alter the activity of Mg^2+^ efflux pathways [3].

### 7.5. Bone

Approximately 60% of total body magnesium resides in bone, predominantly in the hydroxyapatite crystal lattice, where it substitutes for calcium and modulates crystal structure. Tsao et al. [58] investigated the role of SLC41A1 in mesenchymal stromal cells (osteoblast precursors), showing that SLC41A1 knockdown promoted mineralisation and attenuated the inhibitory effect of magnesium on osteogenesis. Bone Mg^2+^ incorporation is thought to depend on the plasma Mg^2+^ concentration and osteoblast transport activity [1,58].

### 7.6. Formulation-Dependent Tissue and Clinical Findings

Several animal studies report formulation-dependent tissue magnesium profiles [59,60,61]. Such findings are relevant to biological differences among formulations, but a tissue concentration difference is not itself evidence of selective transport of an intact magnesium complex.

Magnesium L-threonate and the brain. Slutsky et al. gave magnesium L-threonate to rats and reported higher total CSF magnesium and improved learning and memory, with associated hippocampal synaptic changes [59]. The measured CSF increase was approximately 7% relative to baseline at day 24, estimated at approximately 15% after accounting for a decline in repeatedly sampled controls. These were CSF, not direct brain-tissue magnesium measurements, and some comparative bioavailability data were described as unpublished. Sun et al. subsequently reported threonate-dependent changes in intraneuronal magnesium and synapse density, with effects involving glucose transporters [62]. These findings suggest a possible ligand contribution but do not identify an intact magnesium–threonate complex crossing the intestinal or brain barriers.

**Magnesium acetyl taurate, glycinate, and other salts.** Uysal et al. compared a single oral dose (400 mg/70 kg elemental magnesium) of magnesium sulphate, oxide, acetyl taurate, citrate, and malate in rats. Magnesium acetyl taurate produced the highest brain magnesium concentrations, magnesium malate the highest serum area under the curve, and oxide and citrate the lowest bioavailability [60]. Ates et al. gave magnesium citrate, malate, acetyl taurate, and glycinate to mice at three doses and measured brain, muscle, and serum magnesium after 24 h. Brain magnesium increased in all groups receiving acetyl taurate, and citrate increased muscle and brain magnesium independently of dose, whereas the tissue response to the other salts, including glycinate, varied with dose [61]. Moisa et al. likewise found organ-specific differences between six magnesium compounds in mice, which followed the dissolution behaviour of the compounds (Section 2) [63]. The findings do not contradict ionic magnesium transport: different systemic exposures or ligand effects could produce different tissue responses while channels still conduct Mg^2+^.

**Critical appraisal.** At first sight, these findings appear to contradict the conclusion that the salt form is irrelevant to organ distribution. On closer examination, they are compatible with the free-ion principle and do not demonstrate transport of intact magnesium complexes, for the following reasons:

(1) None of these studies traced the magnesium atom itself, for example, with ^25^Mg or ^26^Mg stable isotopes, or demonstrated an intact magnesium–ligand complex in plasma, CSF, or tissue. Differences in total tissue magnesium after different salts are therefore most parsimoniously explained by differences in the amount and time course of free Mg^2+^ delivered to the circulation, that is, by differences in solubility and intestinal absorption (Section 2), rather than by organ targeting.
(2) Where an anion-specific effect was investigated mechanistically, it was attributable to the anion acting independently [62]. This is a pharmacological effect of the co-ligand (see Section 10), not evidence that magnesium enters the organ as an intact complex.
(3) The studies were performed in rodents, with small groups, single or short-term dosing, and animals that were not magnesium-deficient. Tissue magnesium was measured as total magnesium in homogenates, which cannot distinguish between free and bound, or intracellular and extracellular (including blood-borne), magnesium. Plasma iMg^2+^ exposure was not matched between groups, so a higher tissue value may simply reflect higher absorption.
(4) The reported effects were modest, for example, a 7–15% increase in CSF magnesium with MgT, and the comparative bioavailability data for several salts in the original MgT study were cited as unpublished [59].
(5) Human data are lacking. A randomised, placebo-controlled trial in 109 healthy adults reported improved memory scores after 30 days of a magnesium L-threonate-based formula that also contained phosphatidylserine and vitamins D, C, and B6, but neither brain nor serum magnesium was measured, and the multi-ingredient design does not permit conclusions on magnesium distribution [64]. To our knowledge, no human study has compared the organ distribution of magnesium after magnesium glycinate, taurate, threonate, or other salts using isotope tracers. Hnilicova et al. [65] used ^31^P magnetic resonance spectroscopy to measure free magnesium in the brain of four siblings with Miyoshi myopathy/dysferlinopathy. Reduced values normalised after one month of oral magnesium citrate (400 mg Mg/day). Because citrate is an endogenous metabolite in blood and brain, this observation suggests that an increase in overall magnesium intake can raise brain free magnesium without a specialised carrier anion.

Taken together, these studies show that different magnesium salts can produce different plasma and tissue magnesium profiles in animals. Whether some anions exert relevant biological effects of their own remains debated. The studies do not, however, identify a mechanism by which the salt form could direct magnesium to a particular organ. Rigorous human studies are needed to settle this question, using equimolar elemental doses, stable-isotope tracers, measurement of plasma iMg^2+^, and non-invasive assessment of tissue magnesium, such as ^31^P magnetic resonance spectroscopy-derived intracellular free Mg^2+^ in brain and muscle.

## 8. The Regulatory Network Governing Whole-Body Magnesium Homeostasis

Systemic Mg^2+^ homeostasis is maintained by a highly integrated network of hormonal, transcriptional, and post-translational regulatory mechanisms. Human genetic studies demonstrate that mutations in several key Mg^2+^ transport proteins produce clinically distinct hypomagnesaemia syndromes, confirming the importance of each component.

Key regulatory inputs include: EGF receptor signaling (upregulates TRPM6 expression in the intestine and kidney); 17β-oestradiol (increases TRPM6 expression); cyclosporine and FK506 (immunosuppressants that downregulate TRPM6, causing renal Mg^2+^ wasting); acid-base status (acidosis reduces TRPM6 expression); aldosterone; insulin; and parathyroid hormone [5,10,41].

In addition to these hormonal inputs, Mg^2+^ transporters are linked by direct protein–protein interactions (Figure 2). Phosphatases of regenerating liver (PRL-1/2) bind to CNNM proteins and inhibit CNNM4-dependent Mg^2+^ efflux, thereby raising intracellular Mg^2+^ [66]. CNNM proteins also bind selectively to TRPM7 and stimulate divalent cation entry through the channel, coupling the efflux and influx machinery [67]. The small GTPase ARL15 interacts with the CBS domains of CNNMs and is required for their complex *N*-glycosylation, which modulates CNNM-dependent Mg^2+^ handling [68]. At the epithelial level, EGF acts as an autocrine/paracrine magnesiotropic hormone: a mutation that impairs basolateral sorting of pro-EGF causes isolated recessive renal hypomagnesaemia through insufficient stimulation of TRPM6 [69]. Insulin increases TRPM6 activity via a PI3K- and Rac1-dependent pathway, linking magnesium homeostasis to glucose metabolism [70]. All these interactions converge on the described transporters that handle free Mg^2+^.

The regulatory mechanisms reviewed respond to magnesium availability, hormones, metabolic state and protein interactions. They do not identify a recognition system for the original chemical label of an ingested supplement.

## 9. Critical Evaluation of Organ-Specificity Claims for Magnesium Supplements

Commercial organ-specificity claims sometimes conflate preferential transport of an intact magnesium complex with differences in tissue magnesium concentration or clinical response. The mechanistic literature reviewed establishes ionic magnesium pathways, but does not establish a transporter-mediated route that recognises an ingested complex and delivers it selectively to a target organ. The observation that plasma or tissue Mg^2+^ levels may rise more rapidly with certain salt forms than others is most plausibly attributable to differences in dissolution rate and gastrointestinal transit, that is, to how quickly free Mg^2+^ ions become available for absorption [16,20,21].

## 10. Discussion

The regulation of magnesium homeostasis is essential for normal physiological function. From an evolutionary perspective, our animal and hominin ancestors consumed magnesium-rich diets, and populations consuming traditional diets still show mean adult intakes of 450–700 mg per day [71]. In this context, a major evolutionary challenge was to prevent magnesium overload. Regulatory pathways therefore evolved to ensure an appropriate magnesium supply through the specific absorption mechanisms described above. Cotransport with peptides or other molecular partners would not, in itself, safeguard against unregulated magnesium uptake. In individuals with severely impaired intestinal epithelial barrier function, as may occur with increased intestinal permeability, colitis, or Crohn’s disease, magnesium complexes might cross the damaged barrier. Such uptake, however, would reflect a pathophysiological rather than a normal physiological process. In contrast to ancestral diets, contemporary diets often provide insufficient magnesium, making increased intake necessary to meet individual requirements.

The evidence reviewed indicates that magnesium crosses intact biological membranes as the free divalent cation Mg^2+^. This principle is consistent with the molecular architecture of all characterised magnesium transport proteins and with the thermodynamics of ion channel selectivity.

The clinical implication is clear. Once magnesium from a supplement has been absorbed into the bloodstream, the original salt form is not known to direct its subsequent distribution to organs. Differences between formulations arise before absorption or from effects of the co-ingested anion. The body does not have a routing system that reads the chemical label of a magnesium supplement and directs it to a specified organ. What tissues receive is free plasma iMg^2+^, regulated by the cell-type-specific expression of transport proteins and by the hormonal and metabolic context of each cell.

This does not mean all magnesium supplements are equal in practice. Meaningful differences exist with respect to: (1) dissolution rate and extent in the gastrointestinal tract, (2) gastrointestinal tolerability (inorganic salts, particularly oxide and sulphate, are more likely to cause osmotic diarrhoea at high doses), (3) the presence of pharmacologically active co-ligands (e.g., citrate, aspartate, orotate, taurine, glycine, malate) that may have independent physiological effects, and (4) elemental magnesium content per unit dose.

Healthcare professionals and consumers should be empowered to evaluate magnesium supplement claims critically. A recent survey among family and internal medicine physicians revealed gaps in knowledge about magnesium supplementation [72]. Education about the transport physiology of Mg^2+^ is needed to dispel myths and enable rational supplement selection.

## 11. Conclusions

This review has presented evidence to establish the following conclusions:

Magnesium salts dissociate in the gastrointestinal tract, and only the free Mg^2+^ ion is absorbed by intact intestinal epithelia.

All characterised mammalian magnesium transport proteins (TRPM6, TRPM7, SLC41A1, SLC41A3, CNNM1–4, MagT1 and MRS2) handle the free Mg^2+^ ion. Their selectivity filters and pore architectures are structurally incompatible with the transport of chelated or complexed magnesium species.

Following intestinal absorption, magnesium enters the bloodstream as free iMg^2+^, which equilibrates with protein-bound and anion-complexed fractions using endogenous plasma ligands. The original supplemental anion is metabolized or excreted independently.

Organ-specific uptake of Mg^2+^ is determined by the cell-type-specific expression and regulation of Mg^2+^ transport proteins, by hormonal signals (EGF, PTH, insulin, aldosterone), and by intracellular Mg^2+^ concentration.

Claims that specific magnesium salt forms selectively deliver magnesium to particular organs (brain, heart, muscle) lack a demonstrated mechanistic basis and are not supported by the established biochemistry of magnesium transport.

Rational magnesium supplementation should be guided by elemental magnesium content, dissolution rate, gastrointestinal tolerability, total daily dose and a sufficient duration of intake. These criteria determine how much free Mg^2+^ becomes available for absorption.

## Figures and Tables

**Table 1:** Distribution of magnesium fractions in human plasma. Adapted from Scarpati et al. [36] and Darragh-Hickey et al. [37].

Fraction	Proportion (~%)	Physiological Role
Free ionised Mg^2+^	~55%	Electrophysiologically active; the substrate for cellular uptake
Protein-bound (primarily albumin)	~30%	Storage/buffer pool; not directly available for cellular transport
Complexed with small anions (citrate, phosphate, carbonate, bicarbonate)	~15%	Weakly bound; in equilibrium with free Mg^2+^

**Table 2:** Mammalian Mg^2+^ transport proteins: localisation, direction of transport, main regulators, and phenotypes of loss of function. As far as characterised, all listed proteins handle the free Mg^2+^ ion. DCT, distal convoluted tubule.

Protein	Localisation	Direction/Mechanism	Main Regulators and Interaction Partners	Phenotype of Loss of Function
TRPM6	Apical membrane of enterocytes and DCT cells	Influx; channel, functional as TRPM6/TRPM7 heteromer	EGF, insulin, 17β-oestradiol (stimulation); intracellular Mg^2+^/MgATP, calcineurin inhibitors, acidosis (inhibition)	Hypomagnesaemia with secondary hypocalcaemia (human)
TRPM7	Plasma membrane, ubiquitous	Influx; channel with kinase domain	Intracellular Mg^2+^/MgATP (inhibition); CNNM binding; growth factors	Embryonic lethality (global knockout, mouse); impaired postnatal mineral absorption (intestinal knockout)
MagT1	Plasma membrane; endoplasmic reticulum (oligosaccharyltransferase complex)	Influx (proposed); role as a direct transporter debated	Not well defined	X-linked immunodeficiency with magnesium defect (XMEN, human)
SLC41A1	Plasma membrane, basolateral in epithelia	Efflux; Na^+^/Mg^2+^ exchange (Na^+^-independent transport also reported)	cAMP-dependent signalling	Renal Mg^2+^ wasting (zebrafish knockdown); normal Mg^2+^ homeostasis (mouse knockout)
SLC41A3	Inner mitochondrial membrane; DCT-specific plasma-membrane isoform	Mitochondrial efflux; uptake and extrusion (DCT isoform)	Not well defined	Reduced serum Mg^2+^ (mouse knockout)
CNNM1–4	Plasma membrane (CNNM2: basolateral DCT; CNNM4: basolateral enterocyte)	Efflux and/or Mg^2+^ sensing	PRL-1/2 (inhibition); ARL15 (N-glycosylation); binding to TRPM7	CNNM2: hypomagnesaemia with seizures and intellectual disability; CNNM4: Jalili syndrome (human)
MRS2	Inner mitochondrial membrane	Mitochondrial uptake; CorA-type channel	Cytosolic Mg^2+^ (inhibition)	Impaired mitochondrial Mg^2+^ uptake
Claudin-2, -7, -12	Tight junctions, small intestine	Paracellular, passive	Luminal pH; proton-pump inhibitors (reduced CLDN7/12 expression)	Reduced paracellular Mg^2+^ transport (Caco-2 model)
Claudin-16, -19	Tight junctions, thick ascending limb	Paracellular reabsorption, passive	Ca^2+^/Mg^2+^-sensing receptor	Familial hypomagnesaemia with hypercalciuria and nephrocalcinosis (human)

## Data Availability

No new data were created or analyzed in this study. Data sharing is not applicable to this article.
